# Erratum

**DOI:** 10.1016/j.cjcpc.2023.05.004

**Published:** 2023-05-13

The publisher regrets that disclosure statements were not included in the published version of the following articles that appeared in previous issues of *CJC Pediatric and Congenital Heart Disease*.

The appropriate disclosure statements are included below.

1. “Canadian Developmental Follow-up Practices in Children With Congenital Heart Defects: A National Environmental Scan” [CJC Pediatric and Congenital Heart Disease, Volume 1, Issue 1, 2022, Pages 3-10] https://doi.org/10.1016/j.cjcpc.2021.11.002

**Disclosures**

M.B-.R. received a tier-2 Canada Research Chair in Brain and Child Development from the Canadian Institute of Health Research. The other authors have no conflicts of interest to disclose.

2. “Reducing Barriers to Optimal Automated External Defibrillator Use: An Elementary School Intervention Study” [CJC Pediatric and Congenital Heart Disease, Volume 1, Issue 1, 2022, Pages 30-36] https://doi.org/10.1016/j.cjcpc.2021.12.002

**Disclosures**

None.

